# A novel gene selection algorithm for cancer classification using microarray datasets

**DOI:** 10.1186/s12920-018-0447-6

**Published:** 2019-01-15

**Authors:** Russul Alanni, Jingyu Hou, Hasseeb Azzawi, Yong Xiang

**Affiliations:** School of Information Technology, Deakin University, Burwood, 3125 VIC Australia

**Keywords:** Gene selection, Gene expression programming, Support vector machine, Microarray cancer dataset

## Abstract

**Background:**

Microarray datasets are an important medical diagnostic tool as they represent the states of a cell at the molecular level. Available microarray datasets for classifying cancer types generally have a fairly small sample size compared to the large number of genes involved. This fact is known as a curse of dimensionality, which is a challenging problem. Gene selection is a promising approach that addresses this problem and plays an important role in the development of efficient cancer classification due to the fact that only a small number of genes are related to the classification problem. Gene selection addresses many problems in microarray datasets such as reducing the number of irrelevant and noisy genes, and selecting the most related genes to improve the classification results.

**Methods:**

An innovative Gene Selection Programming (GSP) method is proposed to select relevant genes for effective and efficient cancer classification. GSP is based on Gene Expression Programming (GEP) method with a new defined population initialization algorithm, a new fitness function definition, and improved mutation and recombination operators. . Support Vector Machine (SVM) with a linear kernel serves as a classifier of the GSP.

**Results:**

Experimental results on ten microarray cancer datasets demonstrate that Gene Selection Programming (GSP) is effective and efficient in eliminating irrelevant and redundant genes/features from microarray datasets. The comprehensive evaluations and comparisons with other methods show that GSP gives a better compromise in terms of all three evaluation criteria, i.e., classification accuracy, number of selected genes, and computational cost. The gene set selected by GSP has shown its superior performances in cancer classification compared to those selected by the up-to-date representative gene selection methods.

**Conclusion:**

Gene subset selected by GSP can achieve a higher classification accuracy with less processing time.

## Background

The rapid development of microarray technology in the past few years has enabled researchers to analyse thousands of genes simultaneously and obtain biological information for various purposes, especially for cancer classification. However, gene expression data obtained by microarray technology could bring difficulties to classification methods due to the fact that usually the number of genes in a microarray dataset is very big, while the number of samples is small. This fact is known as the curse of dimensionality in data mining [1–4]. Gene selection, which extracts informative and relevant genes, is one of the effective options to overcome the curse of dimensionality in microarray data based cancer classification.

Gene selection is actually a process of identifying a subset of informative genes from the original gene set. This gene subset enables researchers to obtain substantial insight into the genetic nature of the disease and the mechanisms responsible for it. This technique can also decrease the computational costs and improve the cancer classification performance [5, 6].

Typically, the approaches for gene selection can be classified into three main categories: filter, wrapper and embedded techniques [6, 7]. The filter technique exploits the general characteristics of the gene expressions in the dataset to evaluate each gene individually without considering classification algorithms. The wrapper technique is to add or remove genes to produce several gene subsets and then evaluates these subsets by using the classification algorithms to obtain the best gene subset for solving the classification problem. The embedded technique is between the filter and wrapper techniques in order to take advantage of the merits of both techniques. However, most of the embedded techniques deal with genes one by one [8], which is time consuming especially when the data dimension is large such as the microarray data.

Naturally inspired evolutionary algorithms are more applicable and accurate than wrapper gene selection methods [9, 10] due to their ability in searching for the optimal or near-optimal solutions on large and complex spaces of possible solutions. Evolutionary algorithms also consider multiple attributes (genes) during their search for a solution, instead of considering one attribute at a time.

Various evolutionary algorithms [11–19] have been proposed to extract informative and relevant cancer genes and meanwhile reduce the number of noise and irrelevant genes. However, in order to obtain high accuracy results, most of these methods have to select a large number of genes. Chuang et al. [20] proposed the improved binary particle swarm optimization (IBPSO) method which achieved a good accuracy for some datasets but, again, selected a large number of genes. An enhancement of BPSO algorithm was proposed by Mohamad et al. [21] by minimizing the number of selected genes. They obtained good classification accuracies for some datasets, but the number of selected genes is not small enough compared with other studies.

Recently, Moosa et al. [22] proposed a modified Artificial Bee Colony algorithm (mABC). Another study [15] proposed a hybrid method by using Information Gain algorithm to reduce the number of irrelevant genes and using an improved simplified swarm optimization (ISSO) to select the optimal gene subset. These two studies were able to get a good accuracy with small number of selected genes. However, the number of selected genes is still high compared with our method.

In recent years, a new evolutionary algorithm known as Gene Expression Programming (GEP) was initially introduced by Ferreira [23] and widely used in many applications for classification and decision making [24–30]. GEP has three main advantages

- Flexibility, which makes it easy to design an optimal model. In other words, any part of GEP steps can be improved or changed without adding any complexity to the whole process.
- The power of achieving the target that is inspired from the ideas of genotype and phenotype
- Data visualization. It is easy to visualize each step of the GEP and that distinguishes it from many algorithms

These advantages make it easy to use GEP process to create our new gene selection program and simulate the dynamic process of achieving the optimal solution in decision making.

A few studies applied GEP as a feature selection method and obtained some promising results [31, 32] which encourage us to do further research.

GEP algorithm, based on its evolutionary structure, faces some computational problems, when it is applied to complex and high dimensional data such as microarray datasets. Inspired by the above circumstances, to enhance the robustness and stability of microarray data classifiers, we propose a novel gene selection method based on the improvement of GEP. This proposed algorithm is called Gene Selection Programming (GSP). The idea behind this approach is to control the GEP solution process by replacing the random adding, deleting and selection with the systematic gene-ranking based selection. In this paper four innovative operations are presented: attributes/genes selection (initializing the population), mutation operation, recombination operation and a new fitness function. More details of GSP are presented in the Methods section.

In this work, support vector machine (SVM) with a linear kernel serves to evaluate the performance of GSP. For a better reliability we used leave-one-out cross validation (LOOCV). The results were evaluated in terms of three metrics: classification accuracy, number of selected genes and CPU time.

The rest of this paper is organized as follows: The overview of GEP and the proposed gene selection algorithm GSP are presented in the Methods section (section 2). Results section (section 3) provides the experimental results on ten microarray datasets. Discussion section (section 4) presents the statistical analysis and discussion about the experimental results. Finally, Conclusion section (section 5) gives the conclusions and directions of future research.

## Methods

### Gene expression programming

Gene Expression Programming (GEP) algorithm is an evolutionary algorithm. GEP consists of two parts. The first part is characteristic linear chromosomes (genotype), which are composed of one or more genes. Each gene consists of a head and a tail. The head may contain functional elements like {Q, +, −, ×, /} or terminal elements, while the tail contains terminals only. The terminals represent the attributes in the datasets. In this study, sometimes we use the term attribute to represent the gene in microarray dataset to avoid the possible confusion between the gene in microarray datasets and the gene in GEP chromosome. The size of the tail (t) is computed as t = h (n-1) + 1, where h is the head size, and n is the maximum number of parameters required in the function set. The second part of GEP is a phenotype which is a tree structure also known as expression tree (ET). When the representation of each gene in the chromosome is given, the genotype is established. Then the genotype can be converted to the phenotype by using specific languages invented by the GEP author.

**Fig. 1:**
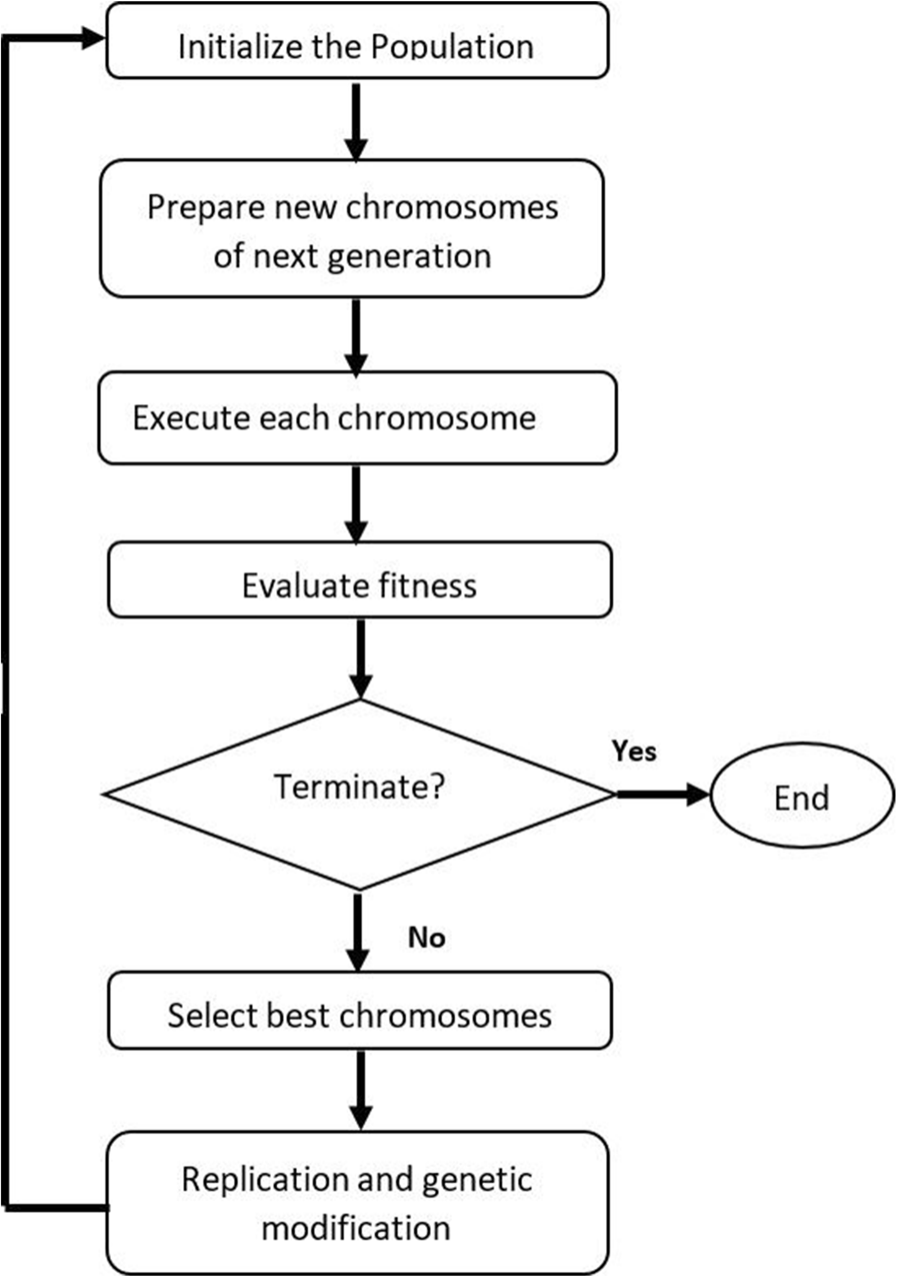
The flowchart of the GEP modelling

GEP process has four main steps: initialize the population by creating the chromosomes (individuals), identify a suitable fitness function to evaluate the best individual, conduct genetic operations to modify the individuals to achieve the optimal solution in the next generation, and check the stop conditions. GEP flowchart is shown in Fig. 1.

It is worth mentioning that the GEP algorithm faces some challenging problems, especially the computational efficiency, when it is applied on the complex and high-dimensional data such as a microarray dataset. This motivates us to solve these problems and further improve the performance of the GEP algorithm by improving the evolution process. The details of the proposed gene selection programming (GSP) algorithm, which is based on GEP, for cancer classification are given in the following sub-sections.

### Systematic selection approach to initial GSP population

Initializing population is the first step in our gene selection method for which candidates are constructed from two sets: terminal set (ts) and function set (fs). Terminal set should represent the attributes of the microarray dataset. The question is what attributes should be selected into the terminal set. Selecting all attributes (including the unrelated attributes) will affect the computational efficiency. The best way to reduce the noise from the microarray data is to minimize the number of unrelated genes. There are two commonly used ways to do that: either by identifying a threshold and the genes ranked above a threshold are selected, or by selecting the n-top ranked genes (e.g. top 50 ranked genes). Both ways have disadvantages: defining a threshold suitable for different datasets is very difficult and deciding how many genes should be selected is subjective. To avoid these disadvantages, we use a different technique named systematic selection approach.

The systematic selection approach consists of three steps: rank all the attributes, calculate the weight of each attribute, and select the attributes based on their weight using the Roulette wheel selection method. The details of these steps are shown in the following sub-sections.

#### Attribute ranking

We use the Information Gain (IG) algorithm [33] to rank the microarray attributes. IG is a filter method mainly used to rank and find the most relevant genes [15, 34, 35]. The attributes with a higher rank value have more impact on the classification process, while the attributes with a zero rank value are considered irrelevant. The rank values of all attributes are calculated once and saved in the buffer for later use in the program.

#### Weight calculation

The weight (w) of each attribute (i) is calculated based on Eq (1)

1 $$ {w}_i=\frac{r_i}{sum}\in \left[0,1\right] $$

where $sum={\sum}_i{r}_{i\kern0.5em }\forall i\in ts$ and *r* is the rank value, and ${\sum}_i{w}_i=1$.

The attributes with a higher weight contain more information about the classification.

#### Attribute selection

In our systematic selection approach, we use the Roulette wheel selection method, which is also known as proportionate selection [36], to select the strong attributes (i.e., the attributes with a high weight). With this approach all the attributes are placed on the roulette wheel according to their weight. An attribute with a higher weight has a higher probability to be selected as a terminal element. This approach could reduce the number of irrelevant attributes in the final terminal set. The population is then initialized from this final terminal set (ts) and the function set (fs).

Each chromosome (c) in GSP is encoded with the length of N*(gene_length), where N represents the number of genes in each chromosome (c) and the length of a gene (g) is the length of its head (h) plus the length of its tail (t). In order to set the effective chromosome length in GSP, we need to determine the head size as well as the number of genes in each chromosome (details are in the Results section). The process of creating GSP chromosomes is illustrated in Algorithm 1

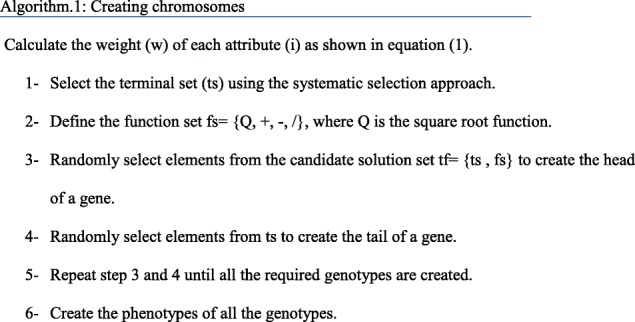



### Fitness function design

The objective of the gene selection method is to find the smallest subset of genes that can achieve the highest accuracy. To this end, we need to define a suitable fitness function for GEP that has the ability to find the best individuals/chromosomes. We define the fitness value of an individual chromosome *i* as follow:

2 $$ {f}_i=2r\ast AC(i)+r\ast \frac{t-{s}_i}{t} $$

This fitness function consists of two parts. The first part is based on the accuracy result *AC*(*i*). This accuracy is measured based on the support vector machine (SVM) classifier using LOOCV.

For example, if chromosome *i* is +/Qa_2_a_1_a_5_a_6_a_3,_ its expression tree (ET) is

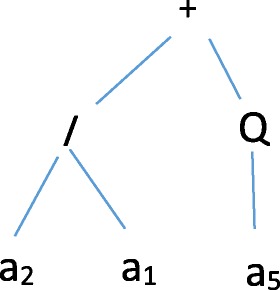



Then, the input values for the SVM classifier are the attributes a_2_, a_1_ and a_5_.

The second part of the fitness function is based on the number of the selected attributes *s*_*i*_ in the individual chromosome and the total number *t* of attributes in the dataset . Parameter *r* is a random value within the range (0.1, 1) giving an importance to the accuracy with respect to the number of attributes. Since the accuracy value is more important than the number of selected attributes in measuring the fitness of a chromosome, we multiply the accuracy by 2r.

### Improved genetic operations

The purpose of the genetic operations is to improve the individual chromosomes towards the optimal solution**.** In this work, we improve two genetic operations as shown below.

#### Mutation

Mutation is the most important genetic operator. It makes a small change to the genomes by replacing an element with another. The accumulation of several changes can create a significant variation. The random mutation may result in the loss of the important attributes, which may reduce the accuracy and increase the processing time.

**Fig. 2:**
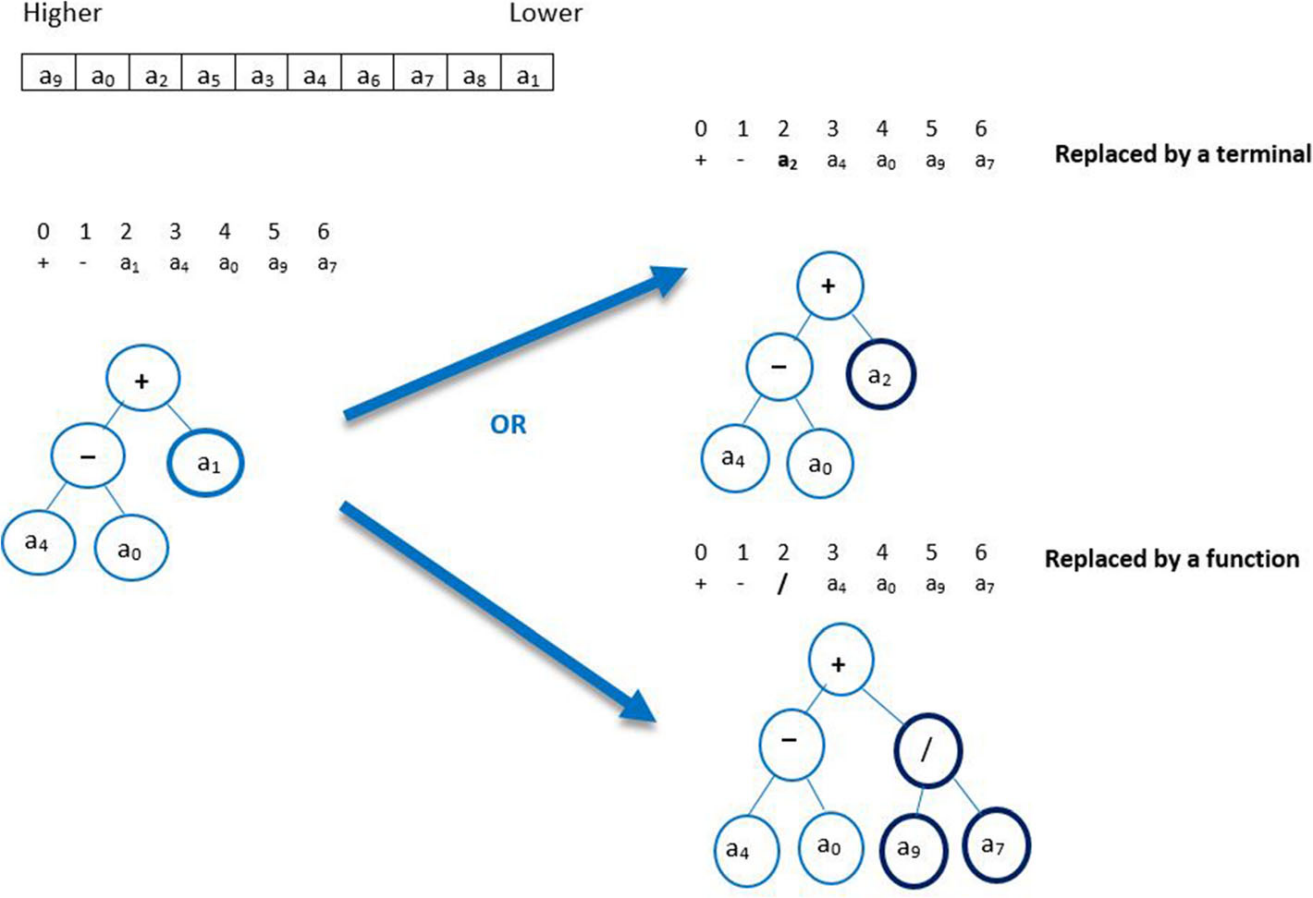
Example of GSP mutation

The critical question of mutation is which attributes are to be added or deleted. Ideally, each deleted terminal/function in the mutation operation should be covered by some other selected terminals/functions. This requirement can be fulfilled by using our method. To clarify the GSP mutation operation, we provide a simple example in Fig. 2.

In the example, the chromosome c has one gene. The head size is 3, so the tail length is h (n-1) + 1=4 and the chromosome length is (3+4) =7. The weight table shows that the attribute with the highest weight in the chromosome is a_9_ and the attribute with the lowest weight is a_1_. With the mutation GSP method selects the weakest terminal *lt* (the terminal with lowest weight) which is a_1_ in our example. There are two options to replace a_1_: the program could select either a function such as (/) or a terminal to replace it. In the latter option, the terminal should have a weight higher than that of a_1_, and the fitness value of the new chromosome c` must be higher than the original one. This new mutation operation is outlined in Algorithm 2.

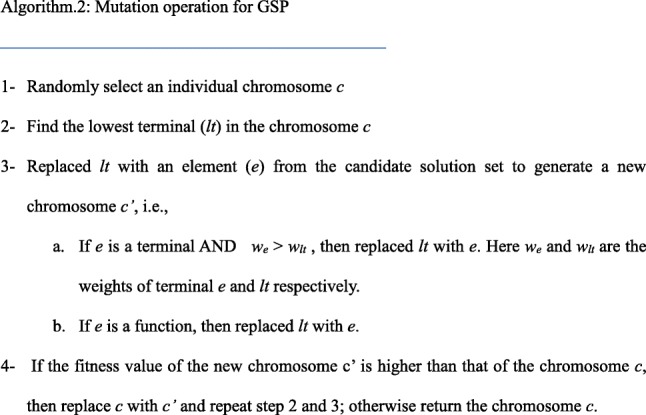



#### Recombination

**Fig. 3:**
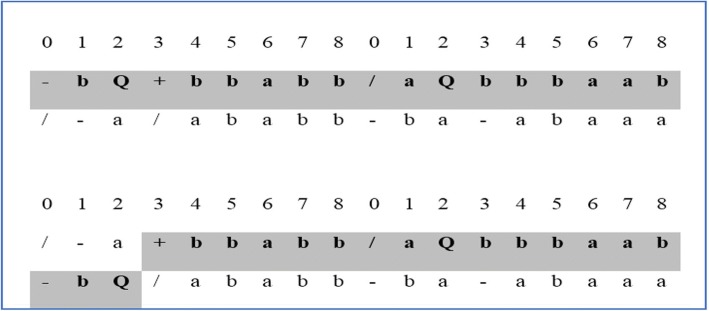
Recombination of 3 elements in gene 1 (from position 0 to 2)

The second operation that we use in our gene selection method is the recombination operation. In recombination, two parent chromosomes are randomly chosen to exchange some material (short sequence) between them. The short sequence can be one or more elements in a gene (see Fig. 3). The two parent chromosomes could also exchange an entire gene in one chromosome with another gene in another chromosome.

**Fig. 4:**
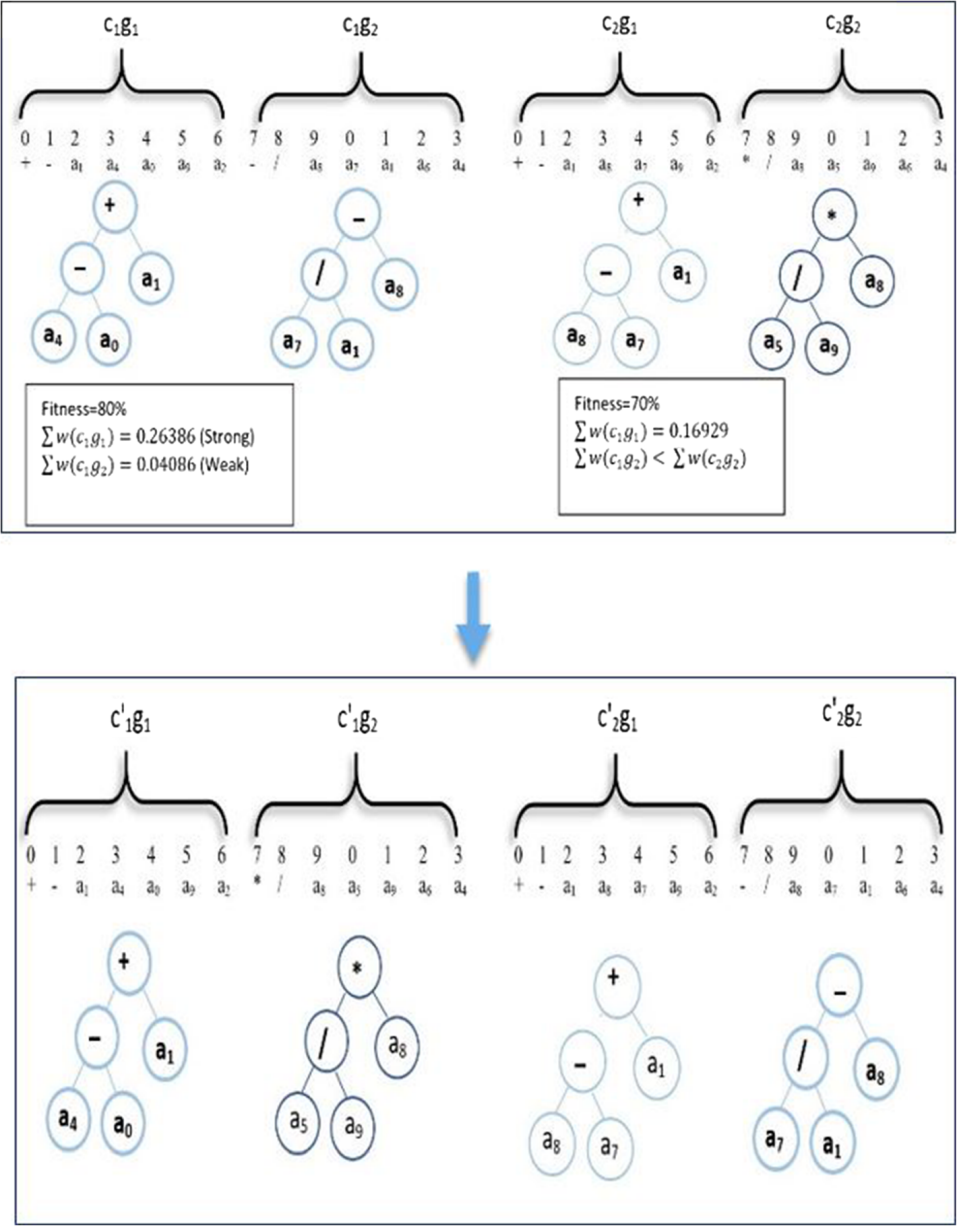
Example for GSP Recombination

In this work, we improve the gene recombination by controlling the exchanging process. Suppose c_1_ and c_2_ are two chromosomes (see Fig. 4). The fitness value of c_1_ = 80% and the fitness value of c_2_ = 70% based on our fitness function (2). We select the “strong” gene (the one with the highest weight summation) from the chromosome that has the lowest fitness value (lc) and exchange it with the “weak” gene (the one with the lowest weight summation) from another chromosome that has the highest fitness value (hc). In general, this process increases the fitness of hc. We repeat the exchange process until we get a new chromosome (hc’) with a higher fitness value than that of both parent chromosomes. The hc` has a higher probability of being a transcription in the next generation. This idea comes from the gene structure [37].

Based on the above innovative improvements for the GSP method in this section, we present the steps of GSP in Algorithm 3 with pseudocode.

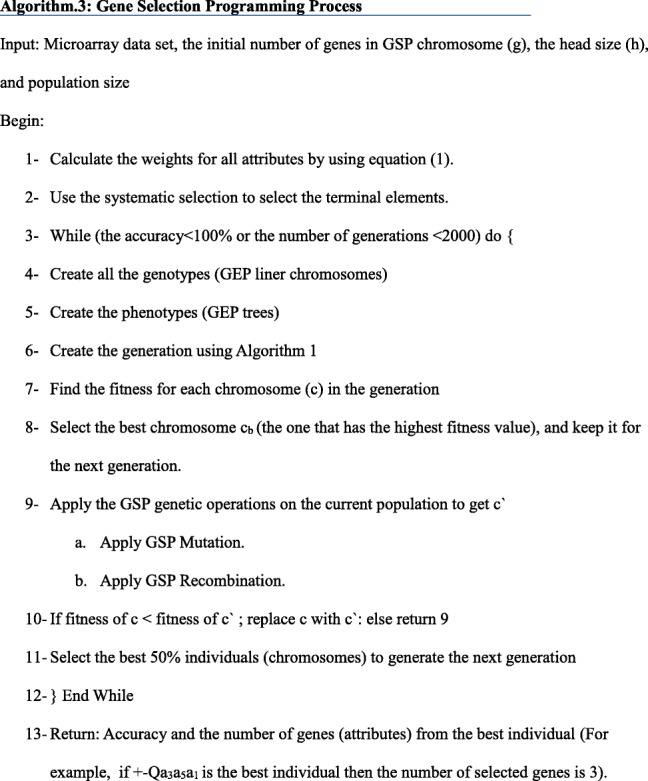



## Results

In this section, we evaluate the performance of GSP method using ten microarray cancer datasets, which were downloaded from http://www.gems-system.org. Table 1 presents the details of the experimental datasets in terms of diverse samples, attributes and classes.

**Table 1:** Description of the experimental datasets

No.	Dataset	Samples	Attributes	Classes	Reference
1	11_Tumors	174	12533	11	[38]
2	9_Tumors	60	5726	9	[39]
3	Brain_Tumor1	90	5920	5	[40]
4	Brain_Tumor2	50	10367	4	[41]
5	Leukemia 1	72	5327	3	[42]
6	Leukemia 2	72	11225	3	[43]
7	Lung_Cancer	203	12600	5	[44]
8	SRBCT	82	2308	4	[45]
9	Prostate_Tumor	102	10509	2	[46]
10	DLBCL	77	5469	2	[47]

Our experimental results contain three parts. Part 1 (Ev.1) evaluated the best setting for GSP based on the number of genes (g) in each chromosome and the head size (h). Part 2 (Ev.2) evaluated the GSP performance in terms of three metrics: classification accuracy, number of selected genes and CPU Time. To guarantee the impartial classification results and avoid generating bias results, this study adopted cross validation method LOOCV to reduce the bias in evaluating their performance over each dataset. Our gene selection results were compared with three gene selection methods using the same classification model for the sake of fair competition. Part 3 (Ev.3) evaluated the overall GSP performance by comparing it with other up-to-date models.

### Ev.1 the best setting for gene and head

To set the best values for the number of genes (g) of each chromosome and the size of the gene head (h) in the GSP method, we evaluated nine different settings to show their effect on the GSP performance results. For g we selected three values 1, 2 and 3, and for each g value we selected three h values: 10, 15 and 20. We increased the values of h by 5 to make it clear to observe the effect of h values on the GSP performance, especially when the effect of increasing h is very slight. For more reliability, we used three different datasets (11_Tumors, Leukaemia 1, Prostate Tumor). The parameters used in GSP are listed in Table 2.

**Table 2:** Parameters used in GSP

Parameter	Setting
Function set	+, -, ÷,Q
Terminal set	Selected informative genes from the microarray dataset using systematic selection.
Number of chromosomes	200
Maximum Number of generations	2000
Genetic operators	
Mutation	0.044
Recombination	0.3

The average results across the three experimental datasets are presented in Table 3. AC_avg_, *N*_avg_ and *T*_avg_ represent the average accuracy, number of selected attributes and CPU time respectively for ten runs, while *AC*_std_, , *N*_std_. and *T*_std._ represent the standard deviation for the classification accuracy, number of selected attributes and CPU time respectively.

**Table 3:** The results of different setting for g and h. Bold font indicates the best results

g	h	*AC*_*avg*_	*AC*_*std*_	*N*_*avg*_	*N*_*std*_	*T*_*avg*_	*T*_*std*_
1	10	87.587	3.423	5.567	2.16	151.1202	0.00594
	15	94.787	2.757	10.067	1.977	154.1243	0.00334
	20	96.317	2.147	11	1.6	157.7917	0.00277
	average	92.897	2.776	8.878	1.912	154.3454	0.004016
2	10	97.453	1.033	11.633	1.637	266.7896	0.00162
	15	**99.543**	**0.183**	**13.267**	0.973	275.1234	0.00146
	20	**99.543**	**0.183**	13.633	0.987	280.1246	0.00149
	average	98.847	0.467	12.844	1.199	274.0125	0.001522
3	10	98.397	0.853	13.133	0.9737	381.0373	0.00445
	15	99.21	0.19	13.3	0.973	382.3714	0.00143
	20	99.21	0.177	13.3	0.973	388.7084	0.00133
	average	98.939	0.407	13.244	0.973	384.039	0.002404

**Fig. 5:**
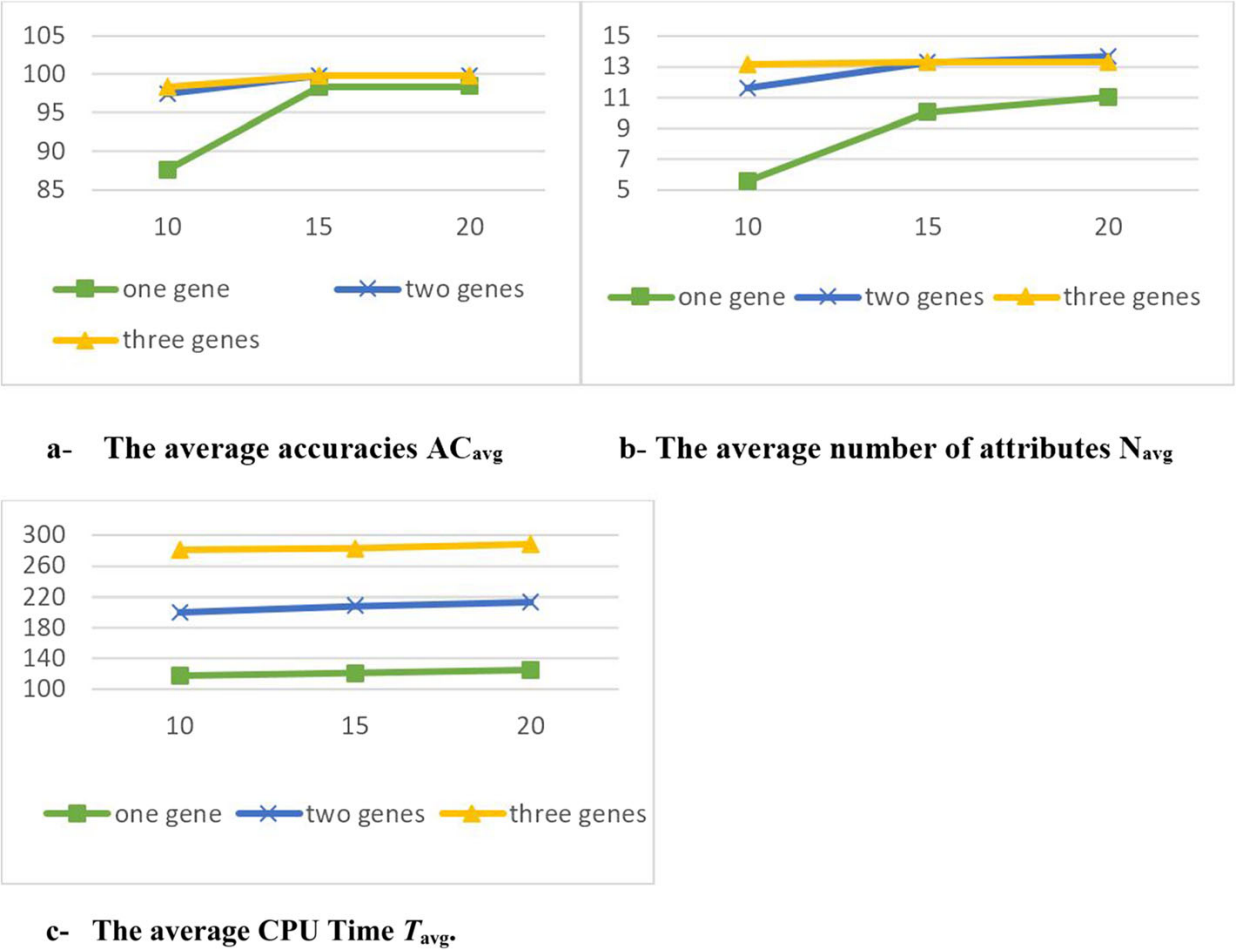
The evaluation values **a** The average accuracies (ACavg). **b** The average number of attributes (Navg ). **c** The average CPU time (Tavg)

Figure 5 shows the evaluation values in terms of AC_avg_, *T*_avg_. and *N*_avg_ for three different numbers of genes in each chromosome.

It is observed from the results in Table 3 that:

1. Comparing g with h: g has a stronger effect on the results than h.
2. Regarding g results: when g was increased, AC_*avg*_*, T*_*avg*_ and *N*_*avg*_ were increased as well (positive relationships). The results of *AC*_*std*_*,, T*_*std.*_ and *N*_*std*_. were decreased when g was increased (negative relationships). The results became stable when the g value was greater than 2.
3. Regarding h results: h has positive relationships with *AC*_*avg*_*, T*_*avg*_ *and N*_*avg*_ and negative relationships with *AC*_*std*_*,, T*_*std.*_ *and N*_*std*_. The results became stable when the h value was over 15.
4. Increasing h values would increase the complexity of the model while the *AC* and *N* results would not show a notable enhancement.
5. The best setting for g and h was 2 and 15 respectively.

### Ev.2: Comparison of the GSP performance with representative gene selection algorithms

In order to evaluate the performance of our GSP algorithm objectively, we first evaluated its performance in terms of three evaluation criteria: classification accuracy (*AC*), number of selected attributes (*N*) and CPU Time (*T*). Then we compared the results with three popular gene selection algorithms named Particle Swarm Optimization (PSO) [48], GEP and GA [49] using the same model for the sake of a fair comparison. The parameters of the comparison methods are listed in Table 4.

**Table 4:** Parameter setting of the competitors

GA		GEP		PSO	
Parameters	Values	Parameters	Values	Parameters	Values
#chromosomes	200	#chromosomes	200	# particles	200
# generations	2000	# generations	2000	# iterations	2000
Crossover rate	0.8	Crossover rate	0.8	Weight (w)	0.8
mutation rate	0.1	mutation rate	0.1	Accelerations c1 and c2	2

The Information Gain algorithm was used in order to filter irrelevant and noisy genes and reduce the computational load for the gene selection and classification methods.

The support vector machine (SVM) with a linear kernel served as a classifier of these gene selection methods. In order to avoid selection bias, the LOOCV was used. Weka software was used to implement the PSO and GA models with default settings, while the GEP model was implemented by using java package GEP4J [50]. Table 5 shows the comparison results of GSP with three gene selection algorithms across ten selected datasets.

**Table 5:** Comparison of GSP with three gene selection algorithms on ten selected datasets. Bold font indicates the best results

Statistics	PSO	GA	GEP	GSP
AC _avg._	96.84	93.14	96.63	**99.92**
AC _std._	3.74	4.78	3.42	**0.06**
SN_avg._	96.49	92.28	96.74	**99.93**
SN_std._	4.05	6.9	3.65	**0.08**
SP_avg._	95.98	93	95.23	**99.93**
SP_std._	5.31	6.03	5.71	**0.09**
AUC_avg._	0.96	0.9	0.96	**1**
AUC_std._	0.05	0.08	0.05	**0**
T _avg_	121	119	235	126
T _std_	30	27	28	38
N _max_.	18.7	549.9	15.7	**7.55**
N _min_.	14.3	462.6	11.5	**6.1**
N _avg_.	16.14	473.5	13.8	**8.16**
N _std_.	10.32	619.44	5.16	**4.86**

The experimental results showed that the GSP algorithm achieved the highest average accuracy result (99.92%) across the ten experimental datasets, while the average accuracies of other models were 97.643%, 97.886% and 94.904% for GEP, PSO and GA respectively.

The standard deviation results showed that GSP had the smallest value (0.342671), while the average standard deviations were3.425399, 3.3534102 and 5.24038421 for GEP, PSO and GA respectively. This means the GSP algorithm made the classification performance more accurate and stable.

The GSP algorithm achieved the smallest number of predictive/relevant genes (8.16), while the average number of predictive genes was 13.8, 16.14 and 473.5 for GEP, PSO and GA respectively.

These evaluation results show that GSP is a promising approach for solving gene selection and cancer classification problems.

CPU Time results showed that GSP took almost half of the time that GEP needed to achieve the best solution. However, the time is still long compared with the PSO and GA methods.

### Ev.3: Comparison of GSP with up-to-date classification models

For more evaluations, we compared our GSP model with up-to-date classification models IBPSO, SVM [14], IG-GA [35], IG-ISSO [15], EPSO [21] and mABC [22]. This comparison was based on the classification result and the number of genes regardless of the methods of data processing and classification. The comparison results on ten datasets are presented in Table 6.

**Table 6:** Comparison of the gene selection algorithms on ten selected datasets. Bold font indicates the best results

		IBPSO	IG-GA	IG-ISSO	EPSO	mABC	SVM	GSP
11_Tumors	AC _avg._	95.06	92.53	95.92	95.4	99.5	**89.08**	**99.88**
	AC _std._	0.3	_____	1.31	0.61	0	**---------**	**0.01**
	N _avg._	240.9	479	19.8	237.7	47.27	**12533**	**17.9**
	N _std._	9.55	____	2.57	9.66	7.79	**-------**	**1.2**
9_Tumors	AC avg.	75.5	85	91.67	75	98.65	**53.33**	**98.88**
	AC _std._	1.58	____	2.48	1.11	0.01	**-------**	**0.02**
	N _avg._	240	52	15.7	247.1	34.73	**5726**	**13.8**
	N _std._	7.95	____	2.2136	9.65	5.54	**--------**	**1.02**
Brain_Tumor1	AC _avg._	92.56	93.33	98	92.11	**100**	90	99.8
	AC _std._	0.54	____	0.88	0.82	**0**	--------	0.31
	N _avg._	11.2	244	10.1	7.5	**16.87**	**5920**	9.2
	N _std._	7.15	____	1.73	2.51	**2.85**	--------	1.5
Brain_Tumor2	AC _avg._	91	88	99.8	92.4	**100**	80	99.9
	AC _std._	0.05	____	0.63	1.27	**0**	--------	0.1
	N _avg._	6.4	489	10.4	6	**10.52**	10367	9.8
	N _std._	1.9	____	1.08	1.83	**1.72**	--------	0.4
Lung_ Cancer	AC _avg._	95.86	95.57	99.41	95.67	100	**95.07**	**100**
	AC _std._	0.53	____	0.45	8.3	0	--------	**0**
	N _avg._	14.9	2101	10.4	8.5	23.31	**12600**	**8.3**
	N _std._	10.57	____	1.08	2.11	5.14	--------	**0.82**
Leukemia1	AC _avg._	100	100	100	100	100	**100**	**100**
	AC _std._	0	____	0	0	0	--------	**0**
	N _avg._	3.5	82	4.6	3.2	5.67	**7129**	**2.9**
	N _std._	0.71	____	0.52	0.63	0.73	--------	**0.73**
Leukemia2	AC avg.	100	98.61	100	100	100	**97.22**	**100**
	AC _std._	0	____	0	0	0	--------	**0**
	N _avg._	6.7	782	4.2	6.8	6.29	**11225**	**4.1**
	N _std._	1.5	____	0.42	2.2	0.98	--------	**0.73**
SRBCT	AC _avg._	100	100	100	99.64	**100**	98.41	100
	AC _std._	0	____	0	0.58	**0**	--------	0
	N _avg._	17.5	56	4.3	14.9	**5.59**	**2308**	4
	N _std._	8.32	____	0.48	13.03	**0.51**	--------	0.67
Prostate	AC _avg._	97.94	96	98.82	97	**100**	93.4	99.87
	AC _std._	0.31	____	0.41	0.62	**0**	--------	0.52
	N _avg._	13.6	343	8.4	6.6	**10.73**	10509	8.2
	N _std._	7.68	____	1.78	2.17	**3.15**	--------	0.79
DLBCL	AC _avg._	100	100	100	100	100	**97.42**	**100**
	AC _std._	0	____	0	0	0	--------	**0**
	N _avg._	6	107	3.9	4.7	4.05	**5469**	**3.5**
	N _std._	1.25	____	0.32	0.82	0.78	--------Ftable	**0.5**

It can be seen from Table 6 that GSP performed better than its competitors on seven datasets (11_Tumors, 9_Tumors, Lung_ Cancer, Leukemia1, Leukemia2, SRBCT, and DLBCL), while mABC had better results on three data sets (Brain_Tumor1, Brain_Tumor2, and Prostate).

Interestingly, all runs of GSP achieved 100% LOOCV accuracy with less than 5 selected genes on the Lung_Cancer, Leukemia1, Leukemia2, SRBCT, and DLBCL datasets. Moreover, over 98% classification accuracies were obtained on other datasets. These results indicate that GSP has a high potential to achieve the ideal solution with less number of genes, and the selected genes are the most relevant ones.

Regarding the standard deviations in Table 6, results that produced by GSP were almost consistent on all datasets. The differences of the accuracy results and the number of genes in each run were very small. For GSP, the highest AC_std_ was 0.52 while the highest N_std_ was 1.5. This means that GSP has a stable process to select and produce a near-optimal gene subset from a high dimensional dataset (gene expression data).

## Discussion

We applied GSP method on ten microarray datasets. The results of GSP performance evaluations show that GSP can generate a subset of genes with a very small number of related genes for cancer classification on each dataset. Across the ten experimental datasets, the maximum number of selected genes is 17 with the accuracy not less than 98.88%.

The performance results of GSP and other comparative models (see Table 6) on Prostate and Brain tumor datasets were not as good as the results on other datasets. This is probably due to the fact that these models concentrated on reducing the number of irrelevant genes, but ignored other issues such as the missing values and redundancy. More effort needs to be made on microarray data processing before applying the GSP model to achieve better results.

The GSP method on datasets 11_Tumors and 9_Tumors achieved relatively lower accuracy results (99.88% and 98.88% respectively) compared with the accuracy results on other datasets. The reason was due to the high number of classes (11 and 9 respectively) which could be a problem to any classification models.

We noticed from GSP performance that when the accuracy increased the number of selected genes and the processing time decreased (negative relationship). This proves that GSP is effective and efficient for gene selection method.

## Conclusions

In this study, we have proposed an innovative gene selection algorithm (GSP). This algorithm can not only provide a smaller subset of relevant genes for cancer classification but also achieve higher classification accuracies in most cases with shorter processing time compared with GEP. The comparisons with the representative state-of-art models on ten microarray datasets show the outperformance of GSP in terms of classification accuracy and the number of selected genes. However, the processing time of GSP is still longer than that of PSO and GA models. Our future research direction is to reduce the processing time of GSP while still keeping the effectiveness of the method.

## Abbreviations

AC: Accuracy
AC_avg_: The average value of accuracy
BPSO: Binary Particle Swarm Optimization
c: Chromosome
ET: Expression Tree
fs: Function set
GA: Genetic Algorithm
GEP: Gene Expression Programming
GSP: Gene Selection Programming
h: Head
IBPSO: Improved Binary Particle Swarm Optimization
ISSO: Improved Simplified Swarm Optimization
LOOCV: Leave-one-out cross validation
mABC: Modified Artificial Bee Colony algorithm
N: Number of genes in each chromosome
Navg: The average number of selected attributes
PSO: Particle Swarm Optimization
r: Rank value
SVM: Support Vector Machine
t: Tail
Tavg: The average value of CPU time
TS: Tabu Search
Ts: Terminal set
w: Weight

## References

[1] Wang H-Q, Jing G-J, Zheng C (2014). Biology-constrained gene expression discretization for cancer classification. Neurocomputing.

[2] Espezua S, Villanueva E, Maciel CD, Carvalho A (2015). A Projection Pursuit framework for supervised dimension reduction of high dimensional small sample datasets. Neurocomputing.

[3] Seo M, Oh S (2013). A novel divide-and-merge classification for high dimensional datasets. Comput Biol Chem.

[4] Xie H, Li J, Zhang Q, Wang Y (2016). Comparison among dimensionality reduction techniques based on Random Projection for cancer classification. Comput Biol Chem.

[5] Tabakhi S, Najafi A, Ranjbar R, Moradi P (2015). Gene selection for microarray data classification using a novel ant colony optimization. Neurocomputing.

[6] Du D, Li K, Li X, Fei M (2014). A novel forward gene selection algorithm for microarray data. Neurocomputing.

[7] Mundra PA, Rajapakse JC (2010). Gene and sample selection for cancer classification with support vectors based t-statistic. Neurocomputing.

[8] Jin C, Jin S-W, Qin L-N (2012). Attribute selection method based on a hybrid BPNN and PSO algorithms. Appl Soft Comput.

[9] Alshamlan H, Badr G, Alohali Y. mRMR-ABC: A Hybrid Gene Selection Algorithm for Cancer Classification Using Microarray Gene Expression Profiling. Biomed Res Int. 2015;2015:604910. 10.1155/2015/604910 PMC4414228 25961028

[10] Alshamlan HM, Badr GH, Alohali YA. The performance of bio-inspired evolutionary gene selection methods for cancer classification using microarray dataset. Int J Biosci, Biochem Bioinformatics. 2014;4:166.

[11] Azzawi H, Hou J, Alanni R, Xiang Y. SBC: A New Strategy for Multiclass Lung Cancer Classification Based on Tumour Structural Information and Microarray Data. *In 2018 IEEE/ACIS 17th International Conference on Computer and Information Science (ICIS),* 2018: 68–73.

[12] Chen K-H, Wang K-J, Tsai M-L, Wang K-M, Adrian AM, Cheng W-C, et al. Gene selection for cancer identification: a decision tree model empowered by particle swarm optimization algorithm. BMC Bioinformatics. 2014;15:1. 10.1186/1471-2105-15-49 PMC3944936 24555567

[13] H. M. Zawbaa, E. Emary, A. E. Hassanien, and B. Parv, "A wrapper approach for feature selection based on swarm optimization algorithm inspired from the behavior of social-spiders," in Soft Computing and Pattern Recognition (SoCPaR), 2015 7th International Conference of, 2015, pp. 25-30.

[14] Mohamad MS, Omatu S, Deris S, Yoshioka M (2011). A modified binary particle swarm optimization for selecting the small subset of informative genes from gene expression data. IEEE Trans Inf Technol Biomed.

[15] Lai C-M, Yeh W-C, Chang C-Y. Gene selection using information gain and improved simplified swarm optimization. Neurocomputing. 2016;19;218:331–8.

[16] D. Karaboga and B. Basturk, "Artificial bee colony (ABC) optimization algorithm for solving constrained optimization problems," in International fuzzy systems association world congress, 2007, pp. 789-798.

[17] Jain I, Jain VK, Jain R (2018). Correlation feature selection based improved-Binary Particle Swarm Optimization for gene selection and cancer classification. Appl Soft Comput.

[18] Pino Angulo A (2018). Gene Selection for Microarray Cancer Data Classification by a Novel Rule-Based Algorithm. Information.

[19] Chuang L-Y, Yang C-H, Yang C-H (2009). Tabu search and binary particle swarm optimization for feature selection using microarray data. J Comput Biol.

[20] Chuang L-Y, Chang H-W, Tu C-J, Yang C-H (2008). Improved binary PSO for feature selection using gene expression data. Comput Biol Chem.

[21] Mohamad MS, Omatu S, Deris S, Yoshioka M, Abdullah A, Ibrahim Z (2013). An enhancement of binary particle swarm optimization for gene selection in classifying cancer classes. Algorithms Mol Biol.

[22] Moosa JM, Shakur R, Kaykobad M, Rahman MS. Gene selection for cancer classification with the help of bees. BMC Med Genet. 2016;9:2–47. 10.1186/s12920-016-0204-7 PMC4980787 27510562

[23] Ferreira C. Gene expression programming in problem solving. In: Soft computing and industry. London: Springer; 2002. p. 635–53.

[24] Azzawi , Hou, J, Xiang Y, Alann R. Lung Cancer Prediction from Microarray Data by Gene Expression Programming. IET Syst Biol. 2016;10(5):168–78. 10.1049/iet-syb.2015.0082 PMC8687242 27762231

[25] Yu Z, Lu H, Si H, Liu S, Li X, Gao C, et al. A highly efficient gene expression programming (GEP) model for auxiliary diagnosis of small cell lung cancer. PloS one. 2015;10:e0125517. 10.1371/journal.pone.0125517 PMC4440826 25996920

[26] Peng Y, Yuan C, Qin X, Huang J, Shi Y (2014). An improved Gene Expression Programming approach for symbolic regression problems. Neurocomputing.

[27] Kusy M, Obrzut B, Kluska J (2013). Application of gene expression programming and neural networks to predict adverse events of radical hysterectomy in cervical cancer patients. Med Biol Eng Comput.

[28] Yu Z, Chen X-Z, Cui L-H, Si H-Z, Lu H-J, Liu S-H (2014). Prediction of lung cancer based on serum biomarkers by gene expression programming methods. Asian Pac J Cancer Prev.

[29] Al-Anni R, Hou J, Abdu-aljabar R, Xiang Y. Prediction of NSCLC recurrence from microarray data with GEP. IET Syst Biol. 2017;11(3):77–85. 10.1049/iet-syb.2016.0033 PMC8687152 28518058

[30] Azzawi H, Hou J, Alanni R, Xiang Y, Abdu-Aljabar R, Azzawi A (2017). Multiclass Lung Cancer Diagnosis by Gene Expression Programming and Microarray Datasets. International Conference on Advanced Data Mining and Applications.

[31] Alsulaiman FA, Sakr N, Valdé JJ, El Saddik A, Georganas ND (2009). Feature selection and classification in genetic programming: Application to haptic-based biometric data. Computational Intelligence for Security and Defense Applications, 2009.

[32] Alanni R, Hou J, Azzawi H, Xiang Y, Lee R (2019). New Gene Selection Method Using Gene Expression Programing Approach on Microarray Data Sets. Computer and Information Science.

[33] Y. Yang and J. O. Pedersen, "A comparative study on feature selection in text categorization," in Icml, 1997, pp. 412-420.

[34] Dai J, Xu Q (2013). Attribute selection based on information gain ratio in fuzzy rough set theory with application to tumor classification. Appl Soft Comput.

[35] Yang C-H, Chuang L-Y, Yang CH (2010). IG-GA: a hybrid filter/wrapper method for feature selection of microarray data. J Med Biol Eng.

[36] Goldberg DE, Deb K (1991). A comparative analysis of selection schemes used in genetic algorithms. Found Genet Algorithms.

[37] Suryamohan K, Halfon MS (2015). Identifying transcriptional cis-regulatory modules in animal genomes. Wiley Interdiscip Rev Dev Biol.

[38] Su AI, Welsh JB, Sapinoso LM, Kern SG, Dimitrov P, Lapp H (2001). Molecular classification of human carcinomas by use of gene expression signatures. Cancer Res.

[39] Staunton JE, Slonim DK, Coller HA, Tamayo P, Angelo MJ, Park J (2001). Chemosensitivity prediction by transcriptional profiling. Proc Natl Acad Sci.

[40] Pomeroy SL, Tamayo P, Gaasenbeek M, Sturla LM, Angelo M, McLaughlin ME (2002). Prediction of central nervous system embryonal tumour outcome based on gene expression. Nature.

[41] Nutt CL, Mani D, Betensky RA, Tamayo P, Cairncross JG, Ladd C (2003). Gene expression-based classification of malignant gliomas correlates better with survival than histological classification. Cancer Res.

[42] Golub TR, Slonim DK, Tamayo P, Huard C, Gaasenbeek M, Mesirov JP (1999). Molecular classification of cancer: class discovery and class prediction by gene expression monitoring. Science.

[43] Armstrong SA, Staunton JE, Silverman LB, Pieters R, den Boer ML, Minden MD (2002). MLL translocations specify a distinct gene expression profile that distinguishes a unique leukemia. Nat Genet.

[44] Bhattacharjee A, Richards WG, Staunton J, Li C, Monti S, Vasa P (2001). Classification of human lung carcinomas by mRNA expression profiling reveals distinct adenocarcinoma subclasses. Proc Natl Acad Sci.

[45] Khan J, Wei JS, Ringner M, Saal LH, Ladanyi M, Westermann F (2001). Classification and diagnostic prediction of cancers using gene expression profiling and artificial neural networks. Nat Med.

[46] Singh D, Febbo PG, Ross K, Jackson DG, Manola J, Ladd C (2002). Gene expression correlates of clinical prostate cancer behavior. Cancer Cell.

[47] Shipp MA, Ross KN, Tamayo P, Weng AP, Kutok JL, Aguiar RC (2002). Diffuse large B-cell lymphoma outcome prediction by gene-expression profiling and supervised machine learning. Nat Med.

[48] Moraglio A, Di Chio C, Poli R (2007). Geometric particle swarm optimisation. European conference on genetic programming.

[49] D. E. Goldberg, "Genetic algorithms in search, optimization and machine learning ‘addison-wesley, 1989," Reading*,* MA, 1989.

[50] J. Thomas, "GEP4J ", ed, 2010.

